# Digitale Integration — Chancen für kleine und mittelständische Unternehmen in Deutschland

**DOI:** 10.1007/s10273-021-2942-1

**Published:** 2021-06-22

**Authors:** Melanie Mesloh

**Affiliations:** Hamburgisches WeltWirtschaftsInstitut gemeinnützige GmbH (HWWI), Oberhafenstraße 1, 20097 Hamburg, Deutschland

**Keywords:** M21, M20, O31

## Abstract

Die Nutzung digitaler Technologien zur Entwicklung innovativer Geschäftsmodelle und interner Prozesse bietet kleinen und mittelständischen Unternehmen die Möglichkeit, auf externe Veränderungen agil zu reagieren. Jedoch zeigt ein internationaler Vergleich der digitalen Wettbewerbsfähigkeit der EU28, dass Deutschland in diesem Bereich Nachholbedarfe hat. Um sich zukünftig im internationalen digitalen Wettbewerb positiv zu positionieren, bedarf es einer gestärkten digitalen Integration der kleinen und mittelständischen Unternehmen, die das Rückgrat der deutschen Wirtschaft bilden. Die bisherige digitale Integration wird untersucht, und die Vorteile und Barrieren der digitalen Integration werden hervorgehoben.

## Abstract (en)

This article describes the level of digital integration of SMEs in Germany. For this purpose, it takes into account the share of selected digital technologies in the business activities of German SMEs. The article also explains advantages and barriers to digital integration and provides an evaluation of selected national framework conditions on the part of SMEs. Finally, the article highlights opportunities and challenges of digital integration in SMEs and presents possible areas for action.

Die Bedeutung von kleinen und mittelständischen Unternehmen (KMU) für die deutsche Wirtschaft ist wohlbekannt. Oft als Motor der deutschen Wirtschaft bezeichnet, repräsentieren KMU über 99 % aller deutschen Unternehmen (Destatis, 2020), beschäftigen mehr als 71 % aller Erwerbstätigen des Landes (KfW, 2020) und erwirtschaften rund 43 % der Bruttowertschöpfung in Deutschland (Destatis, 2020). Unter Rücksichtnahme dieses hohen Stellenwerts für die deutsche Wirtschaft ist insbesondere seit Beginn der COVID-19-Pandemie und der damit einhergehenden wirtschaftlichen Einschränkungen das politische und gesellschaftliche Interesse an den Geschäftsaktivitäten von KMU zunehmend in den Diskussionsfokus gerückt. Im Rahmen der Kontaktbeschränkungen und Schließungen stehen viele traditionell offline geprägte Unternehmen vor neuartigen Herausforderungen. Die digitale Integration bietet die Möglichkeit, diesen Herausforderungen durch die Weiterentwicklung von bestehenden Geschäftsmodellen, sprich der Art und Weise wie die Leistung eines KMU erbracht wird, zu begegnen. In diesem Kontext beschreibt der Begriff digitale Integration in enger Anlehnung an die digitale Transformation den Wandel von zuvor nicht digitalen Leistungen zu digitalen Abläufen (Yoo et al., 2010).

So vielseitig wie die unterschiedlichen Ansprüche und Anwendungsfälle von digitalen Lösungen in den Geschäftsaktivitäten von KMU sind, so vielfältig ist auch deren Auswahl. Neben grundlegenden Lösungen wie z. B. dem Betreiben von Websites und der Nutzung von E-Mail (Castagna et al., 2020) über die Nutzung von Cloud-Computing-Diensten (Kim und Lee, 2015) oder einer aktiven Nutzung von Social-Media-Plattformen (Cesaroni und Consoli, 2015) bis hin zur Gestaltung von digitalen Produktionsschritten im Rahmen von Industrie 4.0, additiver Fertigung oder Big-Data-Analysemethoden (Günther et al., 2017; Loebbecke und Picot, 2015; Rayna und Striukova, 2016) bieten digitale Technologien KMU einen breiten Spielraum, um individuelle Lösungen zu integrieren.

Generell ermöglicht die digitale Integration den KMU, ihre Wettbewerbsfähigkeit im Rahmen von erweiterten Geschäftsaktivitäten zu sichern oder gegebenenfalls auszubauen. Im Hinblick auf die digitale Wettbewerbsfähigkeit von deutschen KMU im europäischen Vergleich sind Chancen und Hemmnisse der digitalen Integration erkennbar. So belegte Deutschland 2019 den zwölften Platz der digitalen Wettbewerbsfähigkeit im EU28-Vergleich (Europäische Kommission, 2020). Wie Abbildung 1 verdeutlicht, weist Deutschland insbesondere einen Verbesserungsbedarf im Bereich der Integration von digitalen Technologien in Geschäftstätigkeiten auf. Hier belegte Deutschland den 18. Platz im EU28-Vergleich (Europäische Kommission,

* Dieser Beitrag ist im Rahmen von „Digital methods, toolbox and trainings for increasing customer-innovation in SMEs (ICIinSMEs)“ entstanden, gefördert durch das Programm Erasmus+ der EU.

**Melanie Mesloh** ist Researcherin am Hamburgischen WeltWirtschaftsInstitut (HWWI) in Hamburg.

Abbildung 1
**Digitale Wettbewerbsfähigkeit im europäischen Vergleich, 2019**

Digital Economy and Society Index

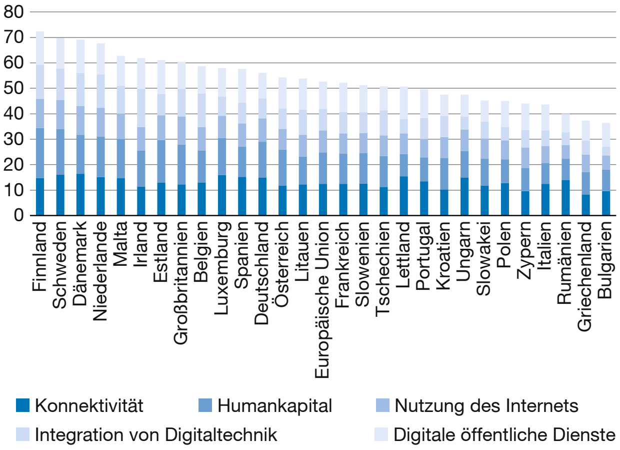

Quelle: Europäische Kommission (2021), Digital Economy and Society Index 2020.

2020). Diese Eindrücke werfen die Frage auf, vor welchen Hürden die Unternehmen stehen.

Zu Beginn kann hervorgehoben werden, dass eine erfolgreiche digitale Integration einen positiven Einfluss auf die unternehmerischen Aktivitäten und die Wettbewerbsfähigkeit von KMU hat (Tarute et al., 2018). Unter anderem dient sie dazu, Prozesse innerhalb der Unternehmen zu verbessern und transparenter zu gestalten. Darüber hinaus werden neue Kommunikationswege eröffnet, wodurch KMU den Austausch z. B. mit Kund:innen oder Beschäftigten ressourcen- und zeitsparend gestalten können (Westerman et al., 2011). Dies führt dazu, dass unternehmerische Risiken gesenkt und die Kundenzufriedenheit gestärkt werden können (Europäische Kommission, 2015). Insbesondere für KMU, die oft unter Restriktionen aufgrund von internen Ressourcenknappheiten (Tarute et al., 2018) leiden, bietet die digitale Integration die Möglichkeit, vorhandene Aktivitäten zu stärken und Raum für die Exploration neuer Möglichkeiten zu bieten. Die digitale Integration kann auf drei Ebenen stattfinden, die in starker Verbindung zueinander stehen. Während die technologische Integration sich auf die Nutzung neuer Technologien wie Social Media oder analytischen Methoden wie Big Data bezieht, zeichnet sich die organisatorische Integration in der Nutzung dieser Technologien zur Erschließung neuer Geschäftsaktivitäten ab. Als dritte Ebene kann die soziale Ebene genannt werden, die sich auf die Digitalisierung des Alltags bezieht (Tarute et al., 2018). Um die Integration von digitalen Lösungen erfolgreich in KMU umzusetzen, bedarf es einer ganzheitlichen Betrachtung der Ebenen, damit mögliche Zusammenhänge und Wechselwirkungen berücksichtigt werden können. Die digitale Integration bietet somit die Möglichkeit, analoge Prozesse von KMU neu zu gestalten und die Wettbewerbsfähigkeit in einer zunehmend digitalen Welt zu erhöhen.

Wenn die Vorteile einer digitalen Integration der KMU realisiert werden, ist es wichtig, die Hürden der KMU bei diesem technologischen Wandel in die Betrachtung einzubeziehen. Eine Hürde von besonderer Bedeutung ist hierbei, dass KMU häufig die finanziellen und zeitlichen Ressourcen sowie die benötigte Expertise fehlen, um neben den laufenden Geschäftsaktivitäten eine digitale Integration durchführen zu können (Stich et al., 2020). Zudem stehen KMU vor der Herausforderung, den internen Wandel zu managen. Dies ist insbesondere durch viele offene Fragen in Hinblick auf z. B. die rechtlichen Anforderungen der Datenspeicherung und -verarbeitung gekennzeichnet. Ebenfalls bremsen bürokratische Anforderungen und mangelhafte Kenntnisse die digitale Integration von KMU (BMWi, 2020; Leifels, 2020; Tarute et al., 2018).

## Status quo der digitalen Integration von KMU

Die Entscheidung von KMU, die digitale Integration umzusetzen, wird von unterschiedlichen internen und externen Faktoren beeinflusst (Tarute et al., 2018). Interne Kommunikationsstrukturen sind hierbei von großer Bedeutung, um den Wandel zu digitalen Geschäftsprozessen zu initiieren und durchzuführen. Sind diese Strukturen gegeben, liegt die Motivation zur digitalen Integration vor allem in der Prozessoptimierung sowie in der Erweiterung und Erschließung neuer Geschäftsbereiche (IUBH, 2019). Insgesamt ist positiv hervorzuheben, dass viele deutsche KMU sich des Wandels bewusst sind. Sie planen mittelfristig die digitale Integration in ihrem Unternehmen umzusetzen (Leifels, 2019).

Diese Einschätzung wird durch die vorliegenden Daten des Statistischen Bundesamts unterstützt. Über die Hälfte der KMU verfügt über eine Internetpräsenz in Form einer Unternehmenswebsite (vgl. Abbildung 2). Eine Vorreiterposition belegen hierbei mit einem Anteil von 93 % KMU mit 50 bis 249 Beschäftigten, gefolgt von 87 % der KMU mit zehn bis 49 Beschäftigten. Schlusslicht bilden die Kleinstunternehmen, von denen 59 % eine Website betreiben. Die IUBH-Studie (2019) unterstreicht dieses Ergebnis, die besagt, das insbesondere bei kleinen Unternehmen der digitale Kundenkontakt ausbaufähig ist. Positiv hervorzuheben ist, dass 69 % der Mitarbeiter:innen in Kleinstunternehmen über einen Internetzugang verfügen. Dies trifft für 57 % der KMU in der mittleren Beschäftigungsgrößenklasse zu. Schlusslicht bilden KMU mit 49 bis 250 Beschäftigten mit einem Anteil von 56 %.

Abbildung 2
**Digitale Integration von KMU, 2020**
in %

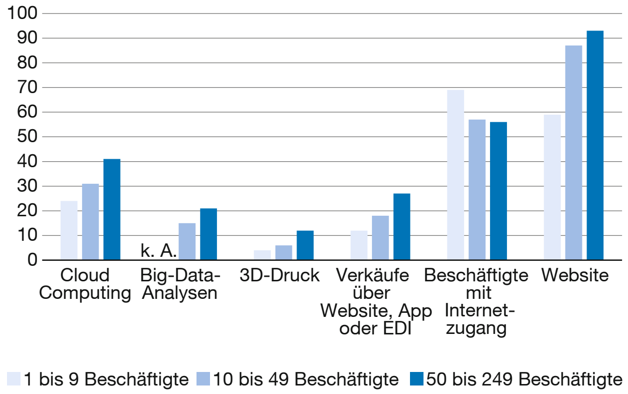

Quelle: Statistisches Bundesamt (2021), IKT-Indikatoren für Unternehmen: Deutschland, Jahre, Beschäftigungsgrößenklassen.

Bei der Betrachtung der digitalen Integration von technologisch und zeitlich anspruchsvolleren digitalen Geschäftsaktivitäten ist erkennbar, dass bei KMU der höheren Beschäftigungsgrößenklassen im Vergleich zu Kleinstunternehmen die digitale Integration weiter vorangeschritten ist. So nutzt knapp ein Viertel der Kleinstunternehmen Cloud Computing Anwendungen, um Bereiche der internen Datenverarbeitungsprozesse durch die Nutzung von Cloud-Lösungen effizienter zu gestalten. Diese digitalen Lösungen sind in 31 % der Unternehmen mit zehn bis 49 Beschäftigten sowie in 41 % der KMU mit 50 bis 249 Beschäftigten integriert.

Laut IUBH-Studie (2019) nutzen KMU die digitale Integration vorrangig zur Optimierung von internen Prozessen, die durch die Implementierung von Cloud Computing in Form von Planungs- und Reportingsoftware sowie durch Software für ein effizientes Customer-Relationship-Management und für interne Kommunikation und Zusammenarbeit unterstützt wird.

Aufgrund fehlender Daten für Kleinstunternehmen kann ein Einblick in die Integration von Big-Data-Analysemethoden für diese Unternehmen nicht erfolgen. Jedoch ist erkennbar, dass die Integration dieser Technologie in mittelgroßen KMU (15 %) und großen KMU (21 %) bereits stattfindet. Im Gegenteil hierzu sind starke Unterschiede in der Nutzung von additiven Fertigungstechnologien im Rahmen von 3D-Druckverfahren erkennbar. Vor allem KMU mit mehr als 50 Beschäftigten nutzten 2020 die Möglichkeiten des 3D-Drucks im Rahmen ihrer Geschäftsaktivitäten. Mit einem Anteil von 12 % ist diese digitale Technologie doppelt so häufig in großen KMU integriert wie in mittelgroßen KMU (6 %). Lediglich 4 % der Kleinstunternehmen gaben an, diese Technologie zu nutzen.

Die Vorteile von E-Commerce, wie z. B. die Möglichkeit, überregionale Märkte zu bedienen und neue Kund:innen zu gewinnen, nutzten 2020 12 % der Kleinstunternehmen in Deutschland. Bei KMU mit zehn bis 49 Beschäftigten liegt der Anteil mit 18 % etwas höher. Bei 27 % der KMU mit 50 bis 249 Beschäftigten ist die Nutzung von E-Commerce bereits Bestandteil des Geschäftsmodells.

Vor dem Hintergrund der rapiden Veränderungen durch die COVID-19-Pandemie 2020 spiegeln die vorliegenden Datensätze nicht alle ausgelösten Entwicklungen wider. Für 2020 sind weitere positive Entwicklungen im Hinblick auf die digitale Integration von KMU zu erwarten. So haben die behördlichen Vorgaben und Aufforderungen zur Kontaktbeschränkung der vergangenen Monate KMU vor neue Herausforderungen gestellt, die als Chancen zur Weiterentwicklung von Geschäftsaktivitäten genutzt werden können. Wie Zimmermann (2020) hervorhebt, haben ca. 43 % der KMU in Deutschland im Zuge der Pandemie Anpassungen an ihrem Geschäftsmodell vorgenommen. Diese Anpassungen fanden sowohl im Bereich des Produkt- und Dienstleistungsangebots als auch in der Weiterentwicklung von vorhandenen Geschäftsmodellen statt.

Ebenfalls kann beobachtet werden, dass viele KMU mithilfe der digitalen Integration im Bereich des Vertriebs auf die Krise reagiert haben. Dies fand insbesondere in der Einführung von Online-Vertriebsmöglichkeiten Anwendung (Zimmermann, 2020). Somit ist anzunehmen, dass der Anteil der KMU, die E-Commerce betreiben, höher liegt, als in den vorliegenden Daten erfasst wurde. Ebenfalls ergab die Sonderuntersuchung des KfW-Mittelstandspanel, dass die Anpassungsbereitschaft der Geschäftsaktivitäten von Kleinstunternehmen (weniger als fünf Beschäftigte) aufgrund der Pandemie deutlich gestiegen ist (Zimmermann, 2020). Insgesamt ist zu erkennen, dass KMU, die auf die Krise mit Anpassungen ihrer geschäftlichen Aktivitäten reagiert haben, eine höhere Resilienz gegenüber externen Einflussfaktoren aufweisen.

### Rahmenbedingungen zur Realisierung von digitaler Integration in KMU

Um die Chancen der digitalen Integration für KMU erfolgreich nutzen zu können, bedarf es externer Rahmenbedingungen sowohl auf institutioneller (z. B. Datensicherheit) als auch auf unternehmerischer und gesellschaftlicher (z. B. Humankapital) Ebene. Bei der Betrachtung der digitalen Infrastruktur in Deutschland ist festzustellen, dass nahezu alle KMU über einen Internetzugang verfügen. Lediglich bei den Kleinstunternehmen gaben 3 % der Unternehmen bzw. 1 % der KMU mit zehn bis 49 Mitarbeiter:innen an, keinen Internetzugang zu besitzen. Somit ist die größte externe Hürde der digitalen Integration genommen (vgl. Abbildung 3). Ein ähnliches Ergebnis ist bei der Verfügbarkeit einer festen Breitbandverbindung zu erkennen. Auch hier ist der Anteil der Kleinstunternehmen mit 89 % am geringsten, gefolgt von der mittleren Größenklasse mit 95 %. 97 % der KMU mit 50 bis 249 Beschäftigten verfügen über eine feste Breitbandverbindung. Die Einschätzungen der Qualität der digitalen Infrastruktur von den KMU deutet jedoch auf ein Verbesserungspotenzial hin. So zeigt sich, dass die verfügbare Geschwindigkeit der Breitbandverbindung für die Tätigkeiten von Kleinstunternehmen am zufriedenstellendsten ist (77 % bewerten die Geschwindigkeit als ausreichend).

Abbildung 3

**Rahmenbedingungen für die digitale Integration in KMU, 2020**

in %

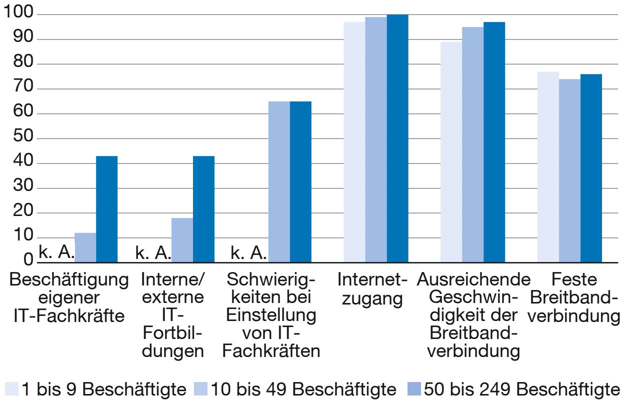

Quelle: Statistisches Bundesamt (2021), IKT-Indikatoren für Unternehmen: Deutschland, Jahre, Beschäftigungsgrößenklassen.

Die Betrachtung der unternehmerischen Rahmenbedingungen zeigt außerdem, dass weniger als die Hälfte der KMU eigene IT-Fachkräfte beschäftigen. Hierbei sind deutliche Unterschiede zwischen den Größenklassen erkennbar. Während 43 % der großen KMU angeben, IT-Fachkräfte zu beschäftigen, gilt dies nur für 12 % der KMU mit zehn bis 49 Mitarbeiter:innen. Den in der Literatur oft diskutierten Fachkräftemangel bestätigten die KMU unabhängig von den Beschäftigungsgrößenklassen. 65 % der KMU gaben an, 2020 Schwierigkeiten bei der Einstellung von IT-Fachkräften gehabt zu haben. Während 43 % der großen KMU diesen Fachkräftemangel durch interne und/oder externe IT-Weiterbildungen ausgleichen konnten, hatten lediglich 18 % der KMU mit zehn bis 49 Beschäftigten diese Gelegenheit, um die benötigte Expertise in das Unternehmen zu holen. Unterschiedliche Weiterbildungsformate können als nachhaltige Möglichkeit zur Aneignung von Grundlagenkenntnissen dienen. Jedoch stehen KMU hier vor der Herausforderung, die Weiterbildungskosten zu finanzieren und gleichzeitig den internen Ausfall des Personals während der Weiterbildungsmaßnahme zu kompensieren (Leifels, 2020). Dies verdeutlicht, dass die digitale Integration der KMU durch mangelnde digitale Kompetenzen der Agierenden behindert wird. Dies führt zu einem hohen Schulungsbedarf, um Grundkompetenzen der Digitalisierung (z. B. Handhabung von digitalen Endgeräten und grundlegender Software) aufzubauen. Außerdem benötigen KMU fortgeschrittene Kompetenzen, um durch die digitale Integration gesammelte Daten zielgerichtet zur Integration in die Geschäftsmöglichkeiten anwenden zu können (Leifels, 2020).

## Fazit

Zusammenfassend kann die Positionierung Deutschlands im Mittelfeld der digitalen Wettbewerbsfähigkeit in der EU28 durch die zuvor beschriebenen Faktoren begründet werden. Die Betrachtung der digitalen Integration von KMU in Deutschland hat verdeutlicht, dass die grundlegenden Rahmenbedingungen weitestgehend erfüllt sind. Ein bedeutendes Hindernis in der Umsetzung der digitalen Lösungen von KMU ist der herrschende Fachkräftemangel. Dieser führt dazu, dass den KMU wichtige Expertise fehlt, um die digitale Integration ressourcen- und zeitschonend zu implementieren. Da Weiter- und Fortbildungsangebote diese Lücke nicht schließen können, stehen insbesondere Kleinstunternehmen vor internen Hürden, um digitale Lösungen in ihre Geschäftsaktivitäten zu integrieren. Aufgrund der kontinuierlich hohen Nachfrage nach IT-Fachkräften stellt der Mangel an Humankapital ein langfristiges Hindernis in der digitalen Integration dar. Darüber hinaus verdeutlichen Studien, dass rechtliche und bürokratische Hürden wie beispielsweise Fragen der Datensicherheit die digitale Integration der KMU verlangsamen. Ebenfalls ist von großer Bedeutung, das Bewusstsein für die digitale Integration der KMU zielgerichtet zu stärken. Um nicht zu stark an der Geschäftskultur deutscher KMU zu rütteln, bietet es sich häufig an, die digitale Integration als Erweiterung bestehender analoger Prozesse zu interpretieren.

Aufgrund der Vielfältigkeit der digitalen Technologien und den damit verbundenen unterschiedlich anspruchsvollen Anforderungen, können auch KMU mit geringen verfügbaren Ressourcen durch die individuelle Nutzung von gezielten digitalen Lösungen ihr Geschäftsmodell neu ausrichten. Diese Neuausrichtung bietet die Möglichkeit, sowohl interne Prozesse durch die Integration von digitalem Projektmanagement oder digitalen Kommunikationskanälen als auch beispielsweise die externe Kommunikation mit Kund:innen durch den gezielten Einsatz von Websites und E-Mail zu stärken. Dies gilt insbesondere für jene Unternehmen, die aufgrund personeller oder finanzieller Restriktionen wenig Ressourcen in die digitale Integration investieren können. Unabhängig von der Unternehmensgröße bietet die Vielseitigkeit der digitalen Integration den KMU die Möglichkeit, unternehmerische Risiken etwa in der Produktentwicklung zu minimieren und ihren Wettbewerbsvorteil zu stärken. Zwar ist vor dem Hintergrund der COVID-19-Krise mit einer zunehmenden digitalen Integration der KMU zu rechnen, jedoch bietet es sich trotzdem an, das Bewusstsein der KMU im Hinblick auf die Chancen einer digitalen Integration z. B. im Rahmen von Aufmerksamkeitskampagnen oder gezielten Trainings zu erhöhen.
